# MMP-9 Knockdown Inhibits Oral Squamous Cell Carcinoma Lymph Node Metastasis in the Nude Mouse Tongue-Xenografted Model through the RhoC/Src Pathway

**DOI:** 10.1155/2021/6683391

**Published:** 2021-03-19

**Authors:** Panpan Yin, Ying Su, Suhong Chen, Jinlin Wen, Feng Gao, Yanlin Wu, Xinyan Zhang

**Affiliations:** Beijing Institute of Dental Research, Beijing Stomatological Hospital & School of Stomatology, Capital Medical University, Beijing, China

## Abstract

Oral squamous cell carcinoma (OSCC) is one of the most common types of cancers in developing countries. A major contributor to the high mortality rate of OSCC is the tendency of oral cancer cells to metastasize to lymph nodes around the head and neck during the early stages of cancer development. Matrix metalloproteinase 9 (MMP-9), an endopeptidase, can degrade the extracellular matrix and basement membrane and plays a key role in tumor invasion and metastasis. *In vitro*, cell migration ability was conducted by scratching assays. We also investigated the interaction abilities between OSCC cells and vascular endothelial cells (ECs) by an adhesion assay and transendothelial migration assay. And we established a BALB/c nude mouse tongue-xenografted metastasis model to investigate the role of MMP-9 and explore its potential underlying mechanism in OSCC growth, lymph node metastasis, and angiogenesis *in vivo*. The results showed that knockdown of MMP-9 could significantly suppress OSCC cell migration, proliferation, interactions between endothelial cells, xenografted tumor growth, and angiogenesis and simultaneously markedly inhibited OSCC cell metastasis to mouse lymphonodi cervicales superficiales, axillary lymph nodes, and even distant inguinal lymph nodes. Mechanistic studies revealed that knockdown of MMP-9 also led to a decreased expression of RhoC, Src, and F-actin by RT-PCR, western blotting, and immunohistochemistry. And the bioinformatic analysis showed that MMP-9, RhoC, and Src mRNA expression was positively and linearly correlated in OSCC on TCGA database. Together, our findings indicated that MMP-9 plays a very important role in OSCC growth, migration, angiogenesis, and lymph node metastasis, and its potential mechanism may be mediated by RhoC and Src gene expression.

## 1. Introduction

Oral squamous cell carcinoma (OSCC) is the most common malignant tumor of the head and neck and accounted for 300,000 cases (2.1% of the world total) and 145,000 deaths (1.8% of the world total) in 2012, according to statistics from the International Agency for Research on Cancer (IARC) [1]. Despite aggressive treatment, the five-year survival rate of OSCC patients is still approximately 50%. The increased mortality of OSCC is related to its aggressive growth pattern with a high degree of local invasiveness and early metastasis to regional lymph nodes [2].

Epithelial-mesenchymal transition (EMT) is considered a transformation process, which enables tumor cells to acquire aggressive characteristics, such as invasiveness and metastasis abilities. In many types of malignancies, such as OSCC, matrix metalloproteinases (MMPs) play a role in tumor microenvironment to induce changes during EMT and promote EMT through invasion and metastasis behavior. Among MMPs, matrix metalloproteinase 9 (MMP-9), the main proteolytic enzyme of MMPs involved in metastasis formation, can degrade various protein components in the extracellular matrix (ECM), disrupt the histological barrier of tumor cell invasion, and result in acceleration of tumor metastasis [3]. MMP-9 is highly expressed in a variety of tumor tissues, including gastric cancer, breast cancer, prostate cancer, osteosarcoma, and non-small-cell lung cancer, and is correlated with clinical classification, lymph node metastasis, and overall survival rates [4–8]. Although the high expression of MMP-9 in clinical studies is considered to be related to tumor invasion, further *in vivo* and *vitro* studies are still needed to confirm the role of MMP-9 in OSCC.

Our preliminary data found that knockdown of MMP-9 could inhibit OSCC cell metastasis in the zebrafish model [9]. To further confirm the role of MMP-9 in metastasis and explore its potential underlying mechanism, we established a BALB/c nude mouse tongue orthotopic OSCC cell xenografted model. Compared with the zebrafish xenografted model, the mouse tongue orthotopic xenografted model has a greater advantage as it allows a better understanding of the effects of MMP-9 gene knockdown in tumor growth, angiogenesis, and local lymph node metastasis and contributes to the further exploration of its underlying mechanism. Specifically, we investigated that knockdown of MMP-9 inhibits the malignant biological behavior of OSCC which may be mediated by RhoC and Src expression.

## 2. Materials and Methods

### 2.1. Establishment of the Knockdown Model of MMP-9 in OSCC Cells

The OSCC cell line CAL27 was obtained from Wuhan University as a gift, and SCC15 was purchased from the American Type Culture Collection (ATCC, Manassas, VA). The cells were cultured in DMEM high glucose or DMEM/F12 medium (Invitrogen Life Science, Carlsbad, CA) supplemented with 10% fetal bovine serum (FBS, HyClone, Logan, UT), 100 U/mL penicillin, and 100 *μ*g/mL streptomycin. The human umbilical vein endothelial cells (HUVECs) were obtained from Peking Union Medical College Hospital and cultured in VascuLife Basal Medium (Lifeline Cell Technology, Frederick, MD). All cells were incubated at 37°C with 5% CO_2_. The stably transfected OSCC cell lines CAL27/MMP-9/shRNA and SCC15/MMP-9/shRNA and their control cells transfected with the control vector were previously established based on HIV lentivirus transfection [9].

### 2.2. Tumor Development in the Nude Mouse Tongue-Xenografted Model

All animal care conditions and experimental protocols were approved by the Animal Ethics Committee of Beijing Stomatological Hospital, Capital Medical University (approval number KQYY-201708-004). The experiments were carried out at the Laboratory Animal Center of Beijing Institute of Dental Research, Beijing Stomatological Hospital, Capital Medical University. Mice were housed and maintained in a specific pathogen-free animal facility in 12 h light/dark cycles, with free access to food and water. Animals did not experience undue suffering at any stage of the experiments. Thirty-five BALB/c nude mice (males, aged 6 weeks, mean weight 19.5 g) were purchased from SPF (Beijing) Biotechnology Co., Ltd. (Beijing, China) and were randomly divided into three groups: group A (11/35): blank control group, group B (12/35): inoculated CAL27/MMP-9/shRNA cells, and group C (12/35): inoculated CAL27/control cells. The nude mice were anesthetized with 1% pentobarbital sodium (Merck, Darmstadt, Germany, 50 mg/kg, IP) and inoculated with the CAL27-transfected OSCC cells (25 *μ*L in PBS, 5 × 10^6^) at the lateral region of the mouse tongue. The body weights of nude mice were measured every three days, and the mice were euthanized 37 days by intraperitoneal injection of pentobarbital sodium (100 mg/kg) following inoculation of OSCC cells. The tongue and lymph node samples were fixed in neutral formalin for hematoxylin and eosin (H&E) and immunohistochemistry staining. A similar tongue orthotopic xenografted model was also established using the SCC15 OSCC cell line (SCC15/MMP-9/shRNA). All experiments were performed in accordance with the institutional guidelines of the Animal Care and Welfare Committee of Beijing Stomatological Hospital, Capital Medical University.

### 2.3. Histopathology and Immunohistochemistry

Tumor tissues were embedded in paraffin wax and cut into 5 *μ*m thick slices, which were then stained with H&E and assessed under an optical microscope (Olympus, BX61, Tokyo, Japan). Immunohistochemistry (IHC) was performed according to the manufacturer's instructions for each antibody. Briefly, after blocking with 10% goat serum, sections were incubated overnight at 4°C with primary antibodies against MMP-9 (ab19906, Abcam, Cambridge, MA, USA,1 : 200), RhoC (ab180785, Abcam, 1 : 200), Src (ab109381, Abcam, 1 : 100), Ki67 (A00052208, DAKO, Denmark, 1 : 200), von Willebrand factor (VWF) (A00042568, DAKO, 1 : 200), and CK (kit-009, Maixin, Fuzhou, China) and then incubated with the respective secondary antibody (kit-5020, Maixin). Immunohistochemical staining was measured with mean optical density (MOD) value by using Image-Pro Plus 6.0 software (MOD = Integral Optical Density (IOD) value/area of the whole).

### 2.4. Immunocytochemistry

We used the methanol to fix cells which were grown on the 13 mm diameter glass for 10 min and used 10% goat serum (ZSBiO, Beijing, China) to block cells for 60 min at 37°C. Then, samples were incubated overnight at 4°C with primary antibodies against RhoC (ab180785, Abcam, 1 : 200) and then incubated with the secondary antibody (kit-5020, Maixin) for 30 min. Mounting medium (Beyotime, Xiamen, China) was used to mount the samples onto slides, and images were acquired using microscopy (Olympus, BX61).

### 2.5. Real-Time PCR

The TRIzol reagent (ComWin Biotech Co., Ltd., Beijing, China) was used to extract the total RNA from the transfected OSCC cells, and the Super RT cDNA Synthesis Kit (ComWin) was used to perform the reverse transcription reaction. PCR was performed using the ULtraSYBR Mixture (Low ROX) (ComWin). The primer sets for human RhoC (catalog number: QRP20382) was provided by GeneCopoeia (Rockville, MD, USA). The primer sets of GAPDH (forward, 5′CATGGGTGTGAACCATGAGAAGTAT-3′; reverse, 5′GACTGTGGTCATGAGTCCTTCCA-3′), MMP-9 (forward, 5′TGTACCGCTATGGTTACACTCG-3′; reverse, 5′GGCAGGGACAGTTGCTTCT-3′), and Src (forward, 5′ATCACCGCAAGAGCTACCAT-3′; reverse, 5′TGACGGTGTCCGAGGAGTTG-3′) were provided by Sangon Biotech Co., Ltd. (Shanghai, China). The PCR results were analyzed using the 2^−ΔΔCt^ method.

### 2.6. Western Blotting

Total protein extracted from transfected OSCC cells was denatured and loaded on 4–20% acrylamide gels (Bio-Rad, Hercules, CA). The proteins were separated by electrophoresis and transferred to a nitrocellulose membrane (Bio-Rad). After blocking with 5% nonfat milk, membranes were incubated with the primary antibodies RhoC (ab180785, Abcam, 1 : 1000), Src (ab109381, Abcam, 1 : 25,000), MMP-9 (ab137867, Abcam, 1 : 600), or GAPDH (M121107, HuaAn Biotechnology, Hangzhou, China, 1 : 2000). Protein bands were then detected by chemiluminescence using the ECL reagent (Thermo Fisher Scientific, Waltham, MA).

### 2.7. Cell Migration Assay

The transfected cells were seeded in 6-well plates (8 × 10^5^/well). After the cells reached confluence, we used a 200 *μ*L pipette tip to make a straight wound in each well and then removed all cell debris with PBS. The cells were cultured in medium containing 2% FBS at 37°C in 5% CO_2_. We used a microscope (Olympus, IX71) with 40x magnification to evaluate the wound closure or filling at 24 h and 48 h.

### 2.8. Adhesion Assay to Endothelial Cells

CAL27/MMP-9/shRNA cells and CAL27/control cells (4.5 × 10^5^/well) were added to confluent HUVECs (6 × 10^3^/well) which were grown in 96-well dishes at 37°C in 5% CO_2_. After 90 min, we used PBS to wash the unadhered cells. And we analyzed the adherent cells with a microplate reader (Molecular Devices, Sunnyvale, CA) at excitation of 580 nm and an emission filter of 630 nm.

### 2.9. Transendothelial Migration Assay

HUVECs were placed into the upper chamber (8 *μ*m pore size; Corning, Corning, NY) of culture plate insert. After the HUVECs had reached confluence, we added (2 × 10^4^/well) CAL27/MMP-9/shRNA cells and CAL27/control cells into the upper chamber at 37°C in 5% CO_2_. After 90 min, we removed the cells which remained on the upper membrane by cotton balls. We counted the cells that had passed through the HUVECs to the lower chamber with fluorescence microscopy (Olympus, IX71).

### 2.10. Immunofluorescence

We used the methanol to fix cells which were grown on the 13 mm diameter glass for 10 min. Then, cells were blocked with 10% goat serum (ZSBiO) for 60 min at 37°C. The F-actin labeled with FITC-phalloidin (3.5 *μ*L of 14 *μ*M F-actin diluted in 500 *μ*L PBS) (Cytoskeleton, Denver, CO) was used to incubate samples for 30 min, and then, the samples were stained with DAPI (Sigma-Aldrich, Saint Louis, MO) for 5 min. Mounting medium (Beyotime) was used to mount the samples onto slides, and images were acquired using a fluorescence microscopy (Olympus, BX61).

### 2.11. Bioinformatic Analysis

To analyze the correlation of MMP-9, RhoC, and Src in OSCC, TCGA data were analyzed (https://www.cancer.gov/about-nci/organization/ccg/research/structural-genomics/tcga). The 341 OSCC raw data were downloaded, and standardized data after log2 transformation of TPM values were used. The standardized data were applied to calculate the MMP-9, RhoC, and Src mRNA level in OSCC. Plots were performed with R v3.6.1.

### 2.12. Statistical Analysis

All experimental data were analyzed using one-way ANOVA or the chi-square test by SPSS Statistics 25.0 (IBM, Armonk, NY). Data were expressed as the mean ± SD of three individual experiments. Statistical significance was set at *P* < 0.05.

## 3. Results

### 3.1. Knockdown of MMP-9 Suppressed Tumor Growth and Proliferation in the Nude Mouse Tongue-Xenografted Model

**Figure 1:**
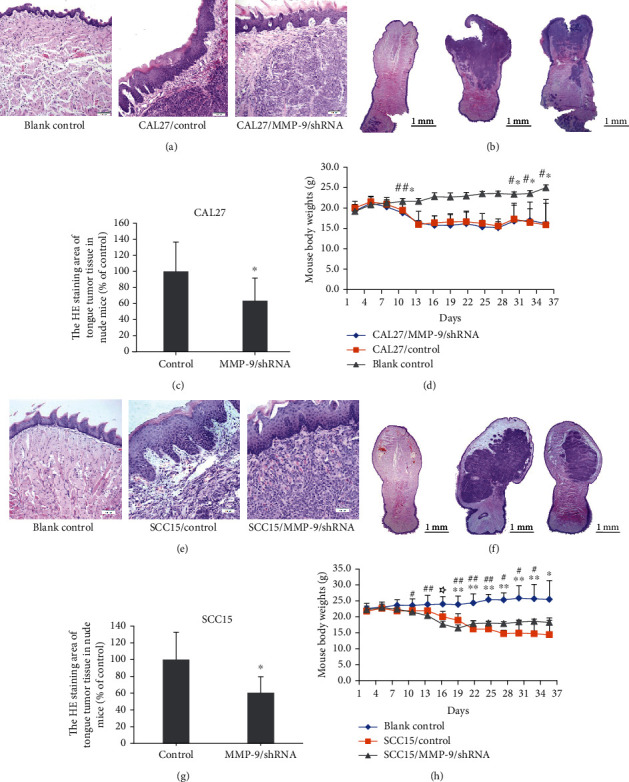
Knockdown of MMP-9 suppresses OSCC growth in the nude mouse tongue-xenografted model. (a, e) H&E staining of the tongue mucosa in CAL27- or SCC15-transfected cells of the nude mouse tongue-xenografted model, respectively. (b, c, e, f) H&E staining of tumor mass in the tongue of nude mice. ^∗^*P* < 0.05, compared with the control group. (d, h) Mouse body weights in CAL27- or SCC15-transfected cells of the nude mouse tongue-xenografted model, respectively. ^∗^*P* < 0.05, control group compared with the blank group; ^∗∗^*P* < 0.01, control group compared with the blank group; ^#^*P* < 0.05, MMP-9/shRNA group compared with the blank group; ^##^*P* < 0.01, MMP-9/shRNA group compared with the blank group; ^☆^*P* < 0.05, MMP-9/shRNA group compared with the control group.

To further confirm the metastasis suppressive effect of MMP-9/shRNA and explore its underlying mechanism *in vivo*, we established a nude mouse tongue-xenografted model with CAL27/MMP-9/shRNA or SCC15/MMP-9/shRNA cells and the respective control cells. And the knockdown efficiency of MMP-9/shRNA-transfected cells has been verified by real-time PCR and western blotting (Figure S1). Figures 1(a) and 1(e) show the histological structure of the mouse tongue mucosa (H&E staining). Compared with the blank control group, injection of CAL27/MMP-9/shRNA or SCC15/MMP-9/shRNA cells led to mild to moderate epithelial dysplasia in the mouse tongue mucosa. However, compared with the vector control cell group, the degree of epithelial cell dysplasia was reduced in the MMP-9/shRNA group (Figures 1(a) and 1(e)). The tumor cell region in the CAL27/MMP-9/shRNA or SCC15/MMP-9/shRNA cell group was reduced compared to that in the control group as shown by H&E staining (Figures 1(b), 1(c), 1(f), and 1(g)). Further, body weight of the blank group mice remained stable (mean body weight was about 22 g in CAL27-transfected cell blank group and 24 g in SCC15-transfected cell blank group), and the body weights of the nude mice injected with CAL27- or SCC15-transfected cells decreased (mean body weight was about 17 g and 18 g, respectively) compared with those of the blank controls, but there was no significant difference between the MMP-9/shRNA and control vector groups (Figures 1(d) and 1(h)).

**Figure 2:**
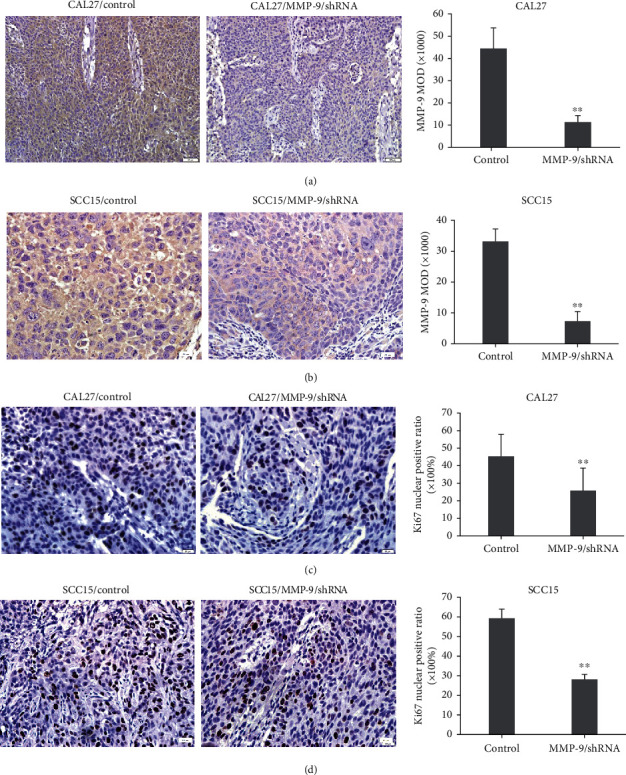
Knockdown of MMP-9 suppresses OSCC cell proliferation in the nude mouse tongue-xenografted model. (a, b) Expression of MMP-9 in CAL27- or SCC15-transfected cells of nude mouse tongue-xenografted tumors by IHC, respectively. (c, d) Cell proliferation is suppressed by MMP-9/shRNA transfection as shown by Ki67-positive cells. All data are shown as the mean ± SD. ^∗∗^*P* < 0.01, compared with the control group.

We also tested the effect of MMP-9 knockdown on cell proliferation in xenograft tumors. Firstly, the expression of MMP-9 in CAL27/MMP-9/shRNA and SCC15/MMP-9/shRNA tongue-xenografted tumors was examined through IHC. The expression of MMP-9 was markedly reduced in the MMP-9/shRNA group compared with the shRNA control group (Figures 2(a) and 2(b)). Furthermore, we evaluated the effect of MMP-9 knockdown on OSCC cell proliferation through Ki67 IHC staining. MMP-9 knockdown decreased CAL27 and SCC15 cell proliferation (Figures 2(c) and 2(d)).

These data indicated that knockdown of MMP-9 could decrease OSCC cell proliferation and tongue-xenografted tumor growth *in vivo*.

### 3.2. Knockdown of MMP-9 Suppressed OSCC Cell Interactions between Endothelial Cells and Xenografted Tumor Angiogenesis

In order to detect the effects of knockdown of MMP-9 on angiogenesis, we investigated the interactions between OSCC cells and vascular endothelial cells by the adhesion assay and transendothelial migration assay *in vitro* firstly and examined the microvessel formation in the nude mouse tongue-xenografted model.

**Figure 3:**
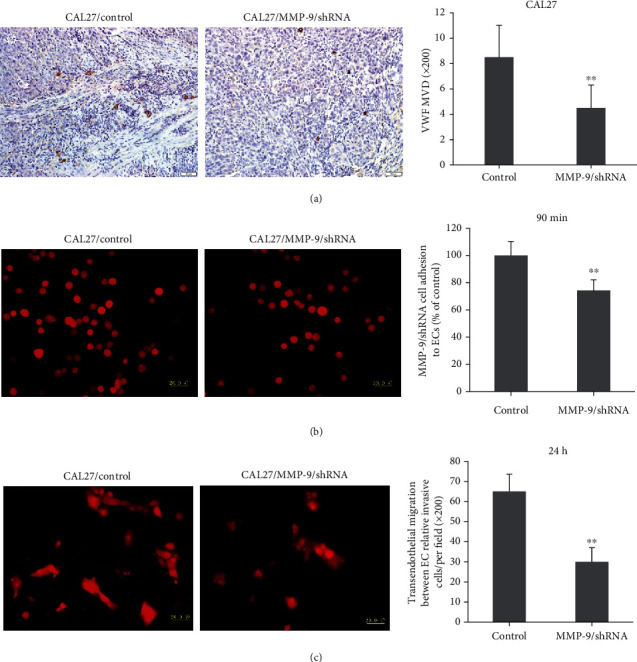
Knockdown of MMP-9 suppressed OSCC cell interactions between endothelial cells and xenografted tumor angiogenesis. (a) MMP-9/shRNA transfection suppresses microvascular density (MVD) by IHC. (b) Knockdown of MMP-9 could decrease cell adhesion to endothelial cells by the adhesion assay. (c) Knockdown of MMP-9 could decrease cell transendothelial migration between endothelial cells. All data are shown as the mean ± SD. ^∗∗^*P* < 0.01, compared with the control group.

Interactions between OSCC cells and vascular endothelial cells during the process of tumor microenvironment were investigated by adding transfected CAL27 cells to confluent HUVECs. After 90 min, we washed off the unattached cells; then, the cells which we observed in the fluorescence microscope were transfected CAL27 cells attached to HUVECs. Briefly, the fluorescence value of cells that adhered to HUVECs was decreased to 74.3% compared to that of the control group through the adhesion assay to endothelial cells (Figure 3(b)).

In addition, we added transfected cells to the HUVECs which attached to the transwell upper chamber. After 24 h culture, we wiped off all cells in the upper chamber; then, the cells that we observed by the fluorescence microscope were transfected cells that passed HUVECs to the lower chamber. The number of cells the passed through the HUVECs within 24 h was markedly reduced from 65.1 per field (×400) of the control group to 30.0 per field (×400) in the MMP-9/shRNA group via the transendothelial migration assay (Figure 3(c)).

Furthermore, we also evaluated the effect of MMP-9 knockdown on OSCC cell angiogenesis through VWF IHC staining in OSCC cell xenografted tumors. The results showed that MMP-9 knockdown decreased OSCC cell angiogenesis (Figure 3(a)).

These data indicated that knockdown of MMP-9 could decrease OSCC cell adhesion to endothelial cells and transendothelial migration between endothelial cells and inhibit OSCC cell xenografted tumor angiogenesis, which was also confirmed in the SCC15 cell line (Figure S2). Thus, MMP-9 is required for OSCC cells to cross the endothelium to complete tumor microenvironment and angiogenesis.

### 3.3. Establishment of the OSCC Cell Nude Mouse Tongue-Xenografted Lymph Node Metastasis Model

Due to the peculiar anatomical structure of the oral cavity, abundant blood supply, and lymphatic reflux, oral cancer cells have strong migration and invasion ability and are especially prone to early metastasis to regional lymph nodes, resulting in low survival rate and poor prognosis of patients. In our murine OSCC cell tongue orthotopic xenografted model, OSCC cells CAL27 and SCC15 transfected with MMP-9 shRNA were able to metastasize to lymph nodes near the oral cavity, to cervical and axillary lymph nodes, and even as far as to the inguinal lymph nodes. However, no metastatic OSCC cells were detected in distant solid organs such as the lungs, liver, or kidneys. As mouse lymph nodes are very small, so we used anti-human cytokeratin pan antibody (CK, a characteristic marker of epithelial cells) to detect the OSSC cells which metastasize into the lymphonodi cervicales superficiales, axillary lymph nodes, and inguinal lymph nodes by IHC.

### 3.4. Knockdown of MMP-9 Inhibited OSCC Cell Migration *In Vitro*

**Figure 4:**
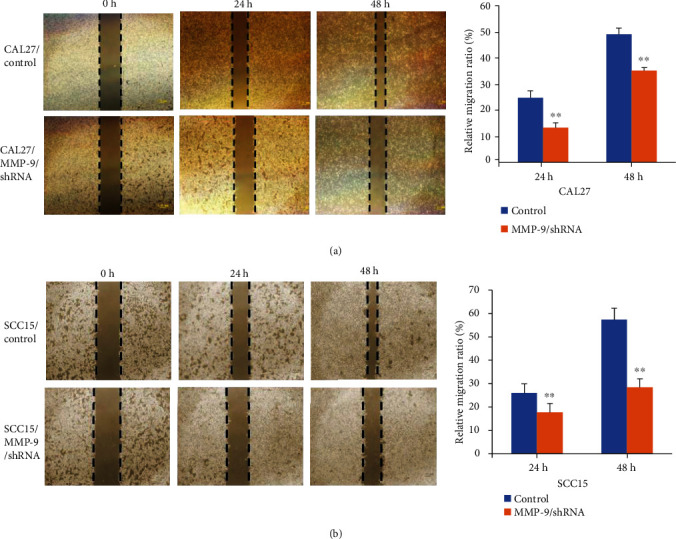
Knockdown of MMP-9 inhibited OSCC cell migration. (a) MMP-9/shRNA transfection inhibited CAL27 cell migration ability by the scratch migration assay. (b) MMP-9/shRNA transfection inhibited SCC15 cell migration ability by the scratch migration assay. ^∗∗^*P* < 0.01, compared with the control group.

In order to verify whether knockdown of MMP-9 affected OSCC cell metastasis ability, we did migration assay *in vitro* firstly. We found that knockdown of MMP-9 could slow down the CAL27 cell relative migration ratio from 24.8% in the control group to 13.2% in the MMP-9/shRNA group at 24 h and from 49.1% to 35.2% at 48 h (Figure 4(a)). Moreover, the cell migration assay was also detected in the SCC15 cell line, and the results were consistent with the CAL27 cell line (Figure 4(b)). These data further demonstrated that the MMP-9 could promote the migration and invasion abilities of OSCC cells.

### 3.5. Knockdown of MMP-9 Inhibited OSCC Cell Metastasis to the Lymphonodi Cervicales Superficiales

**Figure 5:**
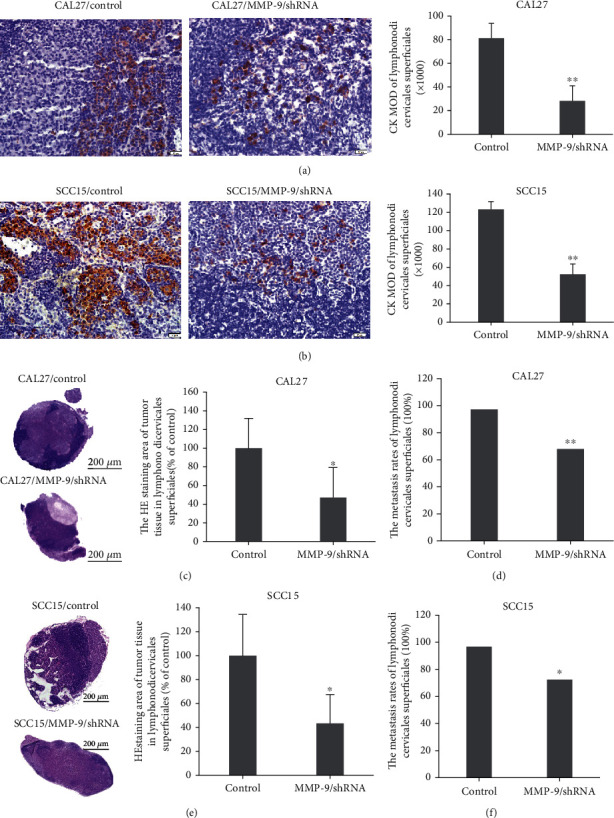
MMP-9 knockdown inhibits OSCC cell metastasis to lymphonodi cervicales superficiales in the nude mouse tongue-xenografted model. (a, b) Expression of CK by IHC in the lymphonodi cervicales superficiales in CAL27- and SCC15-transfected cells of the nude mouse tongue-xenografted model, respectively. (c, e) H&E staining of tumor mass in the lymphonodi cervicales superficiales (independent sample *t*-test). (d, f) The metastasis rates of lymphonodi cervicales superficiales. ^∗^*P* < 0.05, ^∗∗^*P* < 0.01, compared with the control group.

Moreover, in order to detect the metastasis of MMP-9/shRNA-transfected cells in the nude mouse tongue-xenografted model, first we examined lymphonodi cervicales superficiales through cytokeratin IHC and H&E staining. The number of metastatic OSCC cells in the lymphonodi cervicales superficiales of the CAL27/MMP-9/shRNA and SCC15/MMP-9/shRNA groups was significantly decreased compared to that of the control group as shown by the MOD value through IHC staining (Figures 5(a) and 5(b)). H&E staining revealed a metastasis tumor mass in the lymphonodi cervicales superficiales. This metastatic area in the lymphonodi cervicales superficiales was remarkably reduced to 47.2% in the CAL27/MMP-9/shRNA group and reduced to 60.5 in the SCC15/MMP-9/shRNA group compared to the control group, respectively (Figures 5(c) and 5(e)). Moreover, the metastasis rates were also significantly reduced to 68.0% in the CAL27/MMP-9/shRNA group from 97.2% in the control group and reduced to 72.4% in the SCC15/MMP-9/shRNA group from 96.7% in the control group (Figures 5(d) and 5(f)).

### 3.6. Knockdown of MMP-9 Inhibited OSCC Cell Metastasis to the Axillary Lymph Nodes

**Figure 6:**
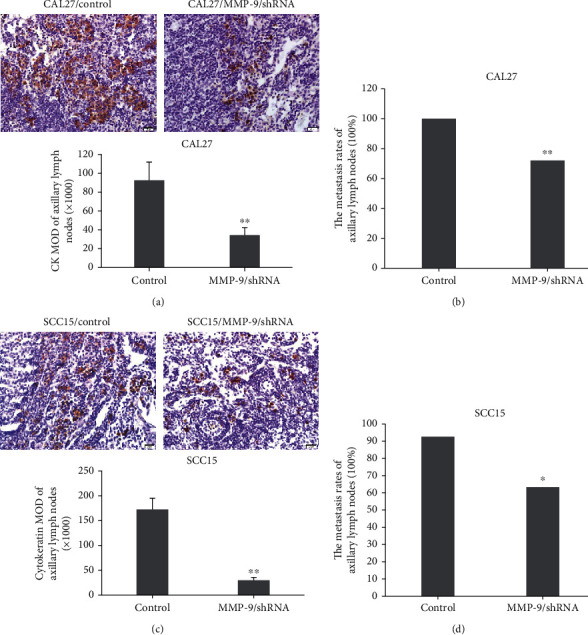
MMP-9 knockdown inhibits OSCC cell metastasis to axillary lymph nodes in the nude mouse tongue-xenografted model. (a, c) Expression of CK by IHC in axillary lymph nodes in CAL27- and SCC15-transfected cells of the nude mouse tongue-xenografted model, respectively (independent sample *t*-test). (b, d) The metastasis rates of axillary lymph nodes. ^∗^*P* < 0.05, ^∗∗^*P* < 0.01, compared with the control group.

Next, we examined mouse axillary lymph node metastasis. The metastasis rates of the axillary lymph nodes were significantly reduced to 72.0% in the MMP-9/shRNA group compared with the control group (100%) (Figure 6(b)). Besides, the number of metastatic OSCC cells in the axillary lymph nodes was significantly decreased from 92.7 in the control to 34.4 in the MMP-9/shRNA group as shown by MOD value through IHC staining (Figure 6(a)). Knocking down MMP-9 gene expression with shRNA also markedly decreased the presence of SCC15 metastatic cells in axillary lymph nodes (Figure 6(c)) and their metastasis rates (Figure 6(d)).

### 3.7. Knockdown of MMP-9 Inhibited OSCC Cell Metastasis to the Inguinal Lymph Nodes

**Figure 7:**
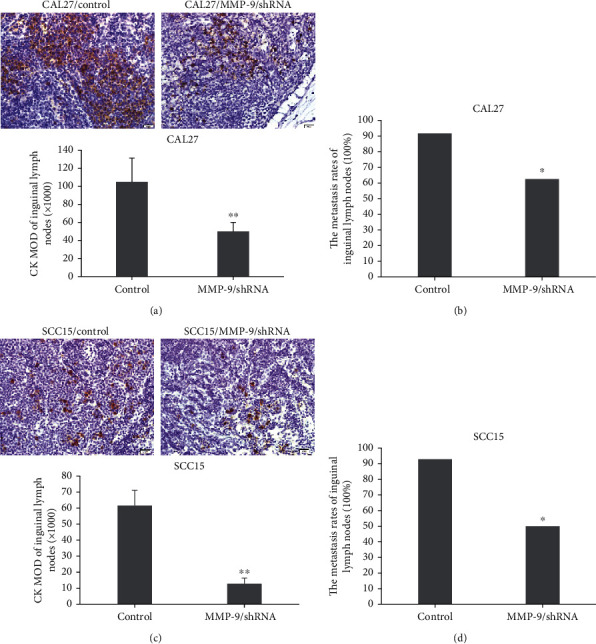
MMP-9 knockdown inhibits OSCC cell metastasis to inguinal lymph nodes in the nude mouse tongue-xenografted model. (a, c) Expression of CK by IHC in inguinal lymph nodes in CAL27- and SCC15-transfected cells of the nude mouse tongue-xenografted model, respectively (independent sample *t*-test). (b, d) The metastasis rates of inguinal lymph nodes. ^∗^*P* < 0.05, ^∗∗^*P* < 0.01, compared with the control group.

We examined the effects of MMP-9 gene knockdown not only in terms of nearby lymph node metastasis but also in terms of distant lymph node inguinal lymph node metastasis. Consistent with the above data, the metastasis rates of the inguinal lymph nodes were remarkably reduced to 62.5% in the MMP-9/shRNA group compared with the control group (91.7%) (Figure 7(b)). Further, the number of metastatic CAL27 cells in the inguinal lymph nodes was also significantly decreased in the MMP-9/shRNA group compared with the control group as shown by MOD values determined by IHC staining (Figure 7(a)). Similar results were also achieved in the SCC15 cell tongue-xenografted model (Figures 7(c) and 7(d)).

Overall, the data indicated that knockdown of MMP-9 inhibited lymph node metastasis in both local and distant regions through inhibition of metastasis rates and the number of metastatic OSCC cells.

### 3.8. Knockdown of MMP-9 Suppressed OSCC Cell RhoC, Src, and F-Actin Expression *In Vitro* and *In Vivo*

**Figure 8:**
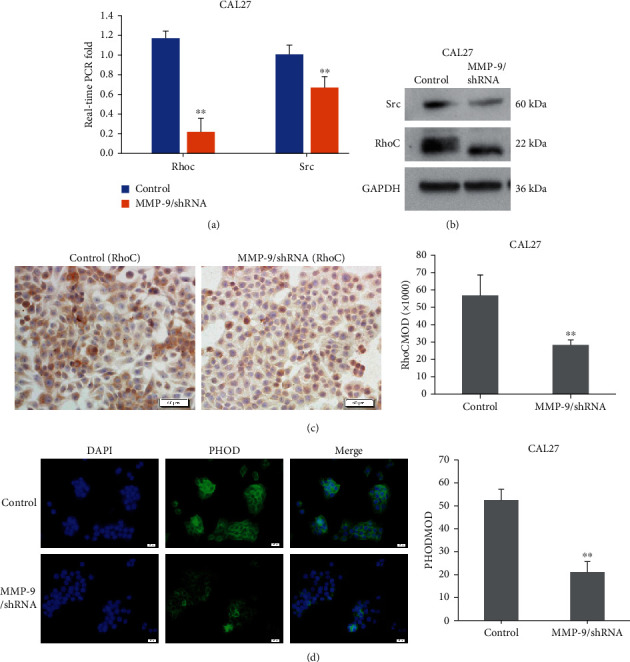
Knockdown of MMP-9 suppresses RhoC, Src, and F-actin expression *in vitro*. (a) RNA expression of RhoC and Src by RT-PCR in CAL27-transfected cells. (b) Protein expression of RhoC and Src by western blotting in CAL27-transfected cells. (c) Knockdown of MMP-9 suppresses RhoC expression by immunocytochemistry. (d) MMP-9/shRNA transfection decreased phalloidin staining by immunofluorescence. All data are presented as the mean ± SD. ^∗∗^*P* < 0.01, compared with the control group.

The above results indicated that knockdown of the MMP-9 gene can effectively inhibit OSCC cell migration, proliferation, and interaction abilities between endothelial cells and suppress distant and near lymph node metastasis. To further investigate the underlying mechanism, we evaluated the expression of RhoC, Src, and F-actin (the key regulators of tumor cell metastasis) in the MMP-9/shRNA-transfected OSCC cells by real-time PCR, western blotting, and IHC. We found that knockdown of MMP-9 could downregulate RhoC and Src RNA and protein expression by real-time PCR (Figure 8(a), Figure S3(a)), western blotting (Figure 8(b), Figure S3(b)), and immunocytochemistry (Figure 8(c), Figure S3(c)) *in vitro*. Moreover, the F-actin expression was also significantly decreased in the MMP-9/shRNA group as compared with the control group as shown by the MOD value through immunofluorescence staining (Figure 8(d), Figure S3(d)).

**Figure 9:**
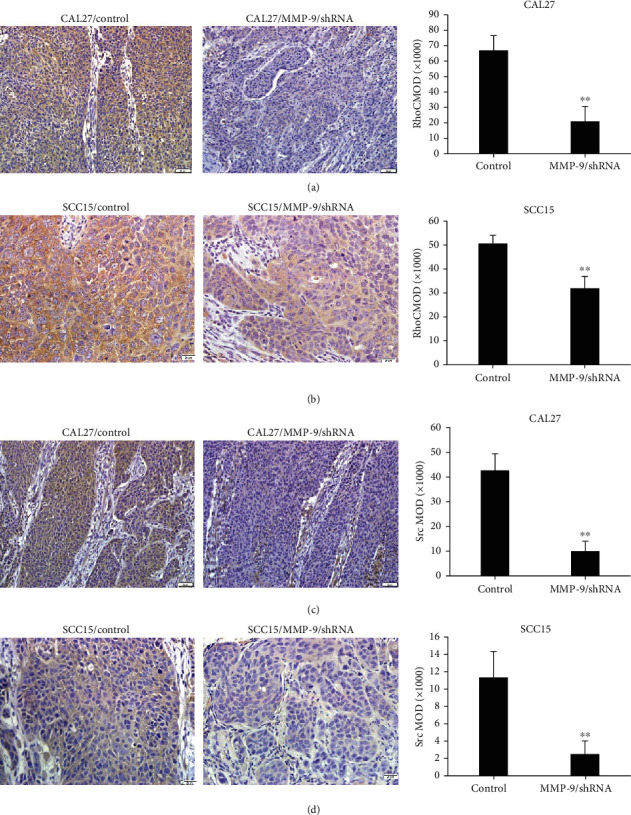
Knockdown of MMP-9 suppresses RhoC and Src expression in tongue-xenografted tumors. (a, b) MMP-9/shRNA transfection inhibits RhoC expression in the cells inoculated with CAL27 or SCC15-transfected cells of the nude mouse tongue-xenografted tumors by IHC, respectively. (c, d) MMP-9/shRNA transfection suppresses Src expression in the nude mouse tongue-xenografted tumors by IHC. All data are presented as the mean ± SD. ^∗∗^*P* < 0.01, compared with the control group.

In the CAL27 or SCC15/MMP-9/shRNA tongue-xenografted tumors, the expression of RhoC and Src was also decreased in the MMP-9/shRNA group compared with the control group as observed by IHC staining (Figures 9(a)–9(d)), which were consistent with the *in vitro* data.

These data indicated that knockdown of MMP-9 expression could downregulate RhoC, Src, and F-actin expression in OSCC.

### 3.9. MMP-9, RhoC, and Src Are Positively and Linearly Correlated in OSCC

**Figure 10:**
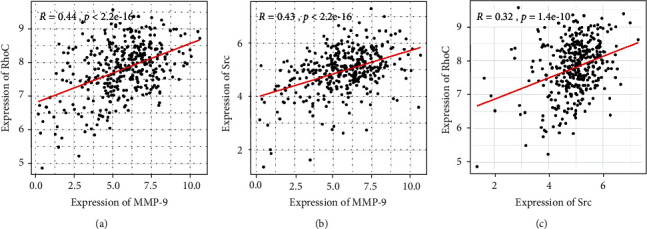
Correlation analysis of MMP-9, RhoC, and Src in OSCC. (a) Correlation analysis of MMP-9 and RhoC. (b) Correlation analysis of MMP-9 and Src. (c) Correlation analysis of RhoC and Src. All mRNA expressions in OSCC were based on TCGA database (including TCGA OSCC samples *n* = 341). *P* < 0.001.

TCGA is the most abundant and authoritative database in tumor research. To further explore the correlation analysis of MMP-9, RhoC, and Src expression, we analyzed the correlation of MMP-9, RhoC, and Src mRNA expression according to the OSCC samples on TCGA database. In OSCC, the positive and linear association of MMP-9 and RhoC (Figure 10(a)), MMP-9 and Src (Figure 10(b)), and RhoC and Src (Figure 10(c)) mRNA expression was observed. Together, our findings indicated that MMP-9 plays a very important role in OSCC invasion and lymph node metastasis, and its potential mechanism may be mediated by RhoC and Src gene expression.

## 4. Discussion

MMP-9 plays a key role in cancer development and progression, which is related to tumor metastasis in many types of malignancies [10–14]. Moreover, deletion of the MMP-9 gene has been reported to delay the tumor onset or suppressed tumor progression in many genetically engineered mouse models of cancers [15–19]. These findings are consistent with our data in this study. MMP-9 plays a crucial role in the early stages of tumor invasion, with the primary function of degrading and remodeling the homeostasis of the ECM. The degradation of the ECM is an essential mechanism in tumor metastasis. OSCC is prone to cervical lymph node metastasis, occurring in about 40% of cases, even in the early stages [20]. In these nude mouse tongue-xenografted models, knockdown of MMP-9 could significantly inhibit OSCC cell metastasis to lymphonodi cervicales superficiales, axillary lymph nodes, and even inguinal lymph nodes. Our data confirmed that MMP-9 plays a pivotal role in OSCC progression at the early stages of lymph node metastasis.

Angiogenesis is a major mechanism involved in tumor metastasis. MMP-9 is often expressed in the angiogenic region [21], which can stimulate the release of tumor vascular growth factors and/or activate the deposition of proangiogenic factors (such as VEGF), thereby recruiting vascular endothelial cells [22] and pericytes [23, 24] required for angiogenesis, resulting in angiogenesis in tumors. Our study showed that knockdown of MMP-9 could suppress the interaction of OSCC cells with vascular endothelial cells *in vitro* and inhibit angiogenesis in nude mouse tongue-xenografted tumors. Our study indirectly confirmed the promoting effect of MMP-9 on tumor angiogenesis.

MMP-9 knockdown inhibits oral cancer cell invasion and metastasis not only by inhibiting OSCC cell proliferation, migration, and vascular endothelial cell adhesion and angiogenesis but also by inhibiting other tumor metastasis-related factors, such as RhoC and Src genes. Our further mechanistic studies indicated that knockdown of MMP-9 inhibited RhoC and Src gene RNA and protein expression *in vivo* and *in vitro*. Furthermore, bioinformatic analysis showed that MMP-9, RhoC, and Src mRNA expression were positively and linearly correlated in OSCC on TCGA database. RhoC, a member of the Rho-GTP family, is a key mediator of tumor cell migration and invasion. It has been reported that RhoC overexpression may predict lymph node metastasis and poor prognosis. Other studies have shown that the deletion of RhoC reduces the ability of tumor cells to adhere to endothelial cells and inhibits the ability of tumor cells to induce endothelial cell junction opening [25]. Src family kinases are widely expressed in human cells, and aberrantly activated Src protein can promote cancer cell proliferation and metastasis and influence the reorganization of the cancer cell cytoskeleton. MMP-9 knockdown inhibited not only the expression of Src but also the expression of F-actin, a cytoskeleton protein.

Tumor cell metastasis is a multistep process involving the degradation of the ECM, cell migration and invasion, cancer cell adhesion to endothelial cells, angiogenesis, and metastatic foci formation [26, 27]. Our results provide compelling evidence that knockdown of MMP-9 has a tumor suppressor function in OSCC, and in particular, MMP-9 exerts a definite inhibitory effect on lymph node metastasis in mice. The potential mechanisms of MMP-9 may relate to the inhibition of a multistep process involving multiple pathways, such as RhoC and Src signaling pathways. Although we identified RhoC, Src, and F-actin as potential targets of MMP-9, further studies are still needed to clarify how MMP-9 regulates its downstream gene expression cascade.

## 5. Conclusions

In summary, our study implied that knockdown of MMP-9 could significantly suppress the malignant biological behavior of OSCC cells, such as migration, metastasis abilities, and interaction between ECs via multiple pathways or mechanisms. Moreover, knockdown of MMP-9 could markedly suppress OSCC cell xenografted tumor growth, proliferation, lymph node metastasis, and angiogenesis in the nude mouse tongue-xenografted model. And further mechanistic studies found that knockdown of MMP-9 may be mediated by the expression of RhoC and Src signaling pathways.

## Data Availability

All the necessary materials can be found in the text or supplementary materials.

## Abbreviations

MMP-9: Matrix metalloproteinase 9
OSCC: Oral squamous cell carcinoma
EMT: Epithelial-mesenchymal transition
ECs: Vascular endothelial cells
ECM: Extracellular matrix
HUVECs: Human umbilical vein endothelial cells
IHC: Immunohistochemistry
VWF: von Willebrand factor
CK: Anti-human cytokeratin pan.

## Supplementary Materials

Supplementary Materials Figure S1: MMP-9 gene knockdown in OSCC cells. (a) MMP-9/shRNA transfection suppressed MMP-9 mRNA expression by RT-PCR. ^∗∗^*P* < 0.01, compared with the control group. (b) MMP-9 expression at the protein level was detected by western blotting. Figure S2: knockdown of MMP-9 suppressed OSCC cell interactions between ECs and xenografted tumor angiogenesis. (a) MMP-9/shRNA transfection suppresses microvascular density (MVD) by IHC. (b) Knockdown of MMP-9 could decrease cell transendothelial migration between ECs. (c) Knockdown of MMP-9 could decrease cell adhesion to ECs by the adhesion assay. All data are shown as the mean ± SD. ^∗∗^*P* < 0.01, compared with the control group. Figure S3: knockdown of MMP-9 suppresses RhoC, Src, and F-actin expression *in vitro*. (a) RNA expression of RhoC and Src by RT-PCR in SCC15-transfected cells. (b) Protein expression of RhoC and Src by western blotting in SCC15-transfected cells. (c) Knockdown of MMP-9 suppresses RhoC expression by immunocytochemistry. (d) MMP-9/shRNA transfection decreased phalloidin staining by immunofluorescence. All data are presented as the mean ± SD. ^∗∗^*P* < 0.01, compared with the control group.
