# Supplementary material for: MMP-9 Knockdown Inhibits Oral Squamous Cell Carcinoma Lymph Node Metastasis in the Nude Mouse Tongue-Xenografted Model through the RhoC/Src Pathway
Source: Anal Cell Pathol (Amst). 2021 Mar 19;2021:6683391. doi: 10.1155/2021/6683391 (PMC8004385; doi:10.1155/2021/6683391)
Supplement: Supplementary Materials — Figure S1: MMP-9 gene knockdown in OSCC cells. (a) MMP-9/shRNA transfection suppressed MMP-9 mRNA expression by RT-PCR. ∗∗P < 0.01, compared with the control group. (b) MMP-9 expression at the protein level was detected by western blotting. Figure S2: knockdown of MMP-9 suppressed OSCC cell interactions between ECs and xenografted tumor angiogenesis. (a) MMP-9/shRNA transfection suppresses microvascular density (MVD) by IHC. (b) Knockdown of MMP-9 could decrease cell transendothelial migration between ECs. (c) Knockdown of MMP-9 could decrease cell adhesion to ECs by the adhesion assay. All data are shown as the mean ± SD. ∗∗P < 0.01, compared with the control group. Figure S3: knockdown of MMP-9 suppresses RhoC, Src, and F-actin expression in vitro. (a) RNA expression of RhoC and Src by RT-PCR in SCC15-transfected cells. (b) Protein expression of RhoC and Src by western blotting in SCC15-transfected cells. (c) Knockdown of MMP-9 suppresses RhoC expression by immunocytochemistry. (d) MMP-9/shRNA transfection decreased phalloidin staining by immunofluorescence. All data are presented as the mean ± SD. ∗∗P < 0.01, compared with the control group. [file 6683391.f1.docx]

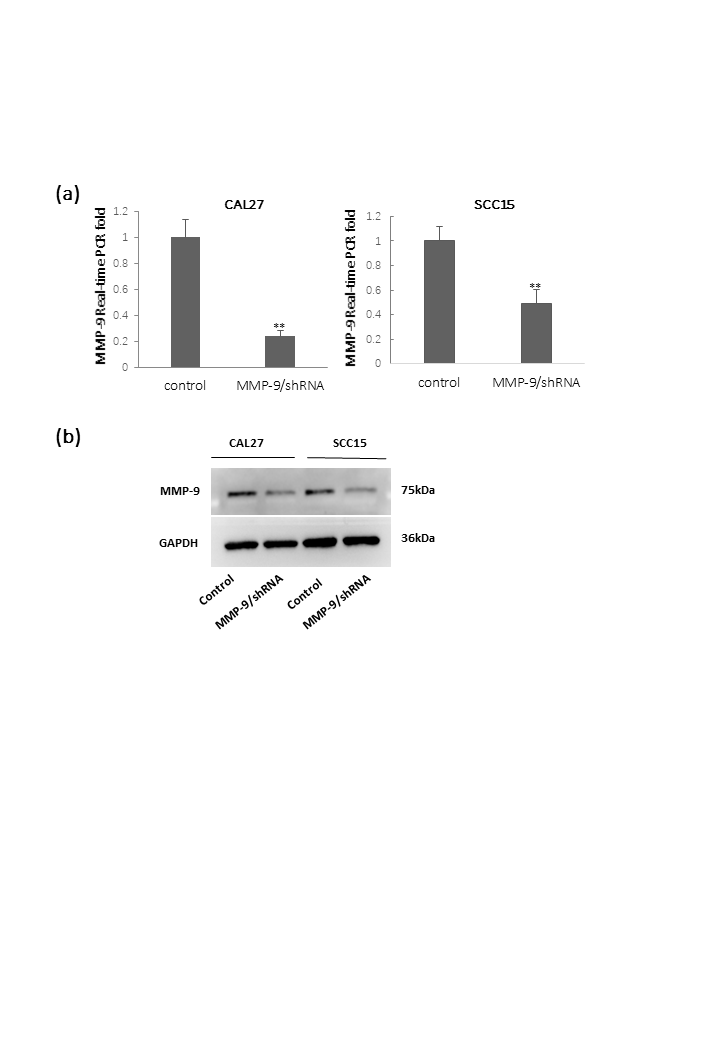


**Figure S1. MMP-9 gene knockdown in OSCC cells.**

(a) MMP-9/shRNA transfection suppressed MMP-9 mRNA expression by RT-PCR. ^**^*P* < 0.01 compared with control group. (b) MMP-9 expression at protein level was detected by western blotting.


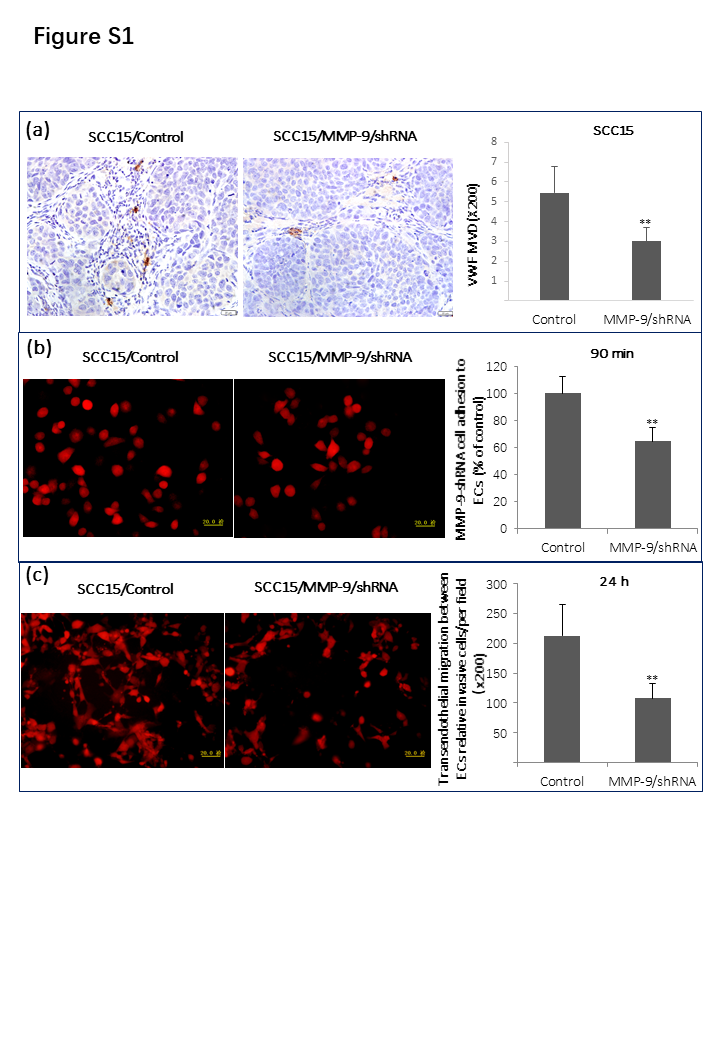


Figure S2. Knockdown of MMP-9 suppressed OSCC cells interactions between ECs and xenografted tumors angiogenesis.

(a) MMP-9/shRNA transfection suppresses microvescular density (MVD) by IHC. (b) Knockdown of MMP-9 could decrease cells transendothelial migration between ECs. (c) Knockdown of MMP-9 could decrease cells adhesion to ECs by adhesion assay. All data are shown as the mean ± SD.^**^*P* < 0.01 as compared with the control group.


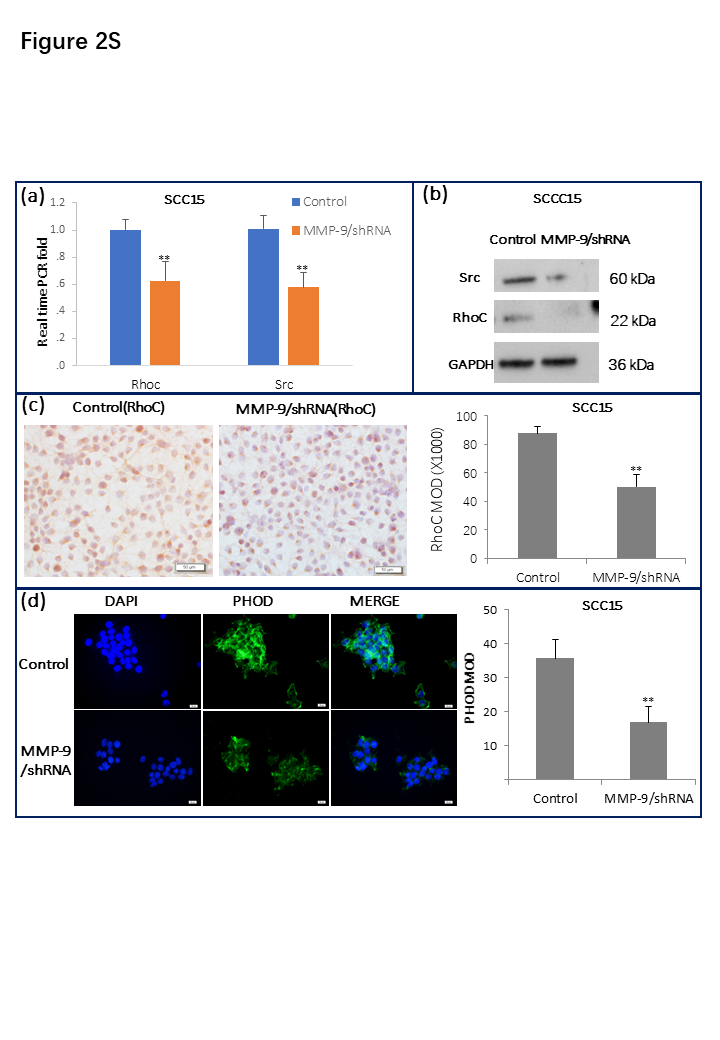


Figure S3. Knockdown of MMP-9 suppresses RhoC, Src, and F-actin expression *in vitro*.

(a) RNA expression of RhoC and Src by RT-PCR in SCC15 transfected cells. (b) Protein expression of RhoC and Src by western blotting in SCC15 transfected cells. (c) Knockdown of MMP-9 suppresses RhoC expression by immunocytochemistry. (d) MMP-9/shRNA transfection decreased phalloidin staining by immunofluorescence. All data are presented as the mean ± SD. ^**^*P* < 0.01 compared with the control group.
